# Recent Advances in Centrifugal Spinning and Their Applications in Tissue Engineering

**DOI:** 10.3390/polym15051253

**Published:** 2023-03-01

**Authors:** Shaik Merkatur Hakim Marjuban, Musfira Rahman, Syeda Sharmin Duza, Mohammad Boshir Ahmed, Dinesh K. Patel, Md Saifur Rahman, Karen Lozano

**Affiliations:** 1 Department of Materials Science and Engineering, Texas A&M University, College Station, TX 77843, USA; 2 Department of Mechanical Engineering, University of Texas Rio Grande Valley, Edinburg, TX 78539, USA; 3 Zachry Department of Civil and Environmental Engineering, Texas A&M University, College Station, TX 77840, USA; 4 Microbiology & Immunology Department, Holy Family Red Crescent Medical College & Hospital, Dhaka 1000, Bangladesh; 5 School of Materials Science and Engineering, Gwangju Institute of Science and Technology, Gwangju 61005, Republic of Korea; 6 Department of Biosystems Engineering, Institute of Forest Science, Kangwon National University, Chuncheon 24341, Republic of Korea; 7 Department of Biomedical Engineering, Texas A&M University, College Station, TX 77843, USA

**Keywords:** centrifugal spinning, jet trajectory, polymer solution, nanofiber, tissue engineering

## Abstract

Over the last decade, researchers have investigated the potential of nano and microfiber scaffolds to promote wound healing, tissue regeneration, and skin protection. The centrifugal spinning technique is favored over others due to its relatively straightforward mechanism for producing large quantities of fiber. Many polymeric materials have yet to be investigated in search of those with multifunctional properties that would make them attractive in tissue applications. This literature presents the fundamental process of fiber generation, and the effects of fabrication parameters (machine, solution) on the morphologies such as fiber diameter, distribution, alignment, porous features, and mechanical properties. Additionally, a brief discussion is presented on the underlying physics of beaded morphology and continuous fiber formation. Consequently, the study provides an overview of the current advancements in centrifugally spun polymeric fiber-based materials and their morphological features, performance, and characteristics for tissue engineering applications.

## 1. Introduction

The popularity of fiber-based composites in tissue engineering can be attributed to their high aspect ratios, high surface areas, and diverse morphologies. A common method called electrospinning, a non-woven fiber creation technology, may create fibers with a diameter of anywhere from hundreds to thousands of nanometers in range, making it one of several possible approaches to achieving such desirable properties [1]. However, electrospinning has various downsides; for example, a low yielding rate, a higher voltage setup, surface charge, and the need for a dielectric solution to facilitate optimal fiber production [2,3,4].The ease of implementation of the centrifugal spinning method has made it a popular choice for addressing these issues. In certain instances, under identical processing conditions, centrifugally spun fiber can exhibit superior mechanical properties compared to electrospun fiber [5]. Nanofiber constructions of any desired form, morphology, or orientation (beaded, textured, or smooth) can be produced using this process. By adjusting the process variables such as concentration viscosity [6,7,8], molecular weight [9,10,11,12], the collector shape [13,14], and other factors [15,16,17], it is possible to influence the fiber homogeneity [18,19], alignments [20,21,22], diameters [15,22,23] and web porosity [24,25,26,27]. Fiber fabrication in centrifugal spinning is not dependent on the conductivity of the solution, hence it may be used with a broad variety of polymer emulsions and suspensions, and it can reach substantially higher production rates than standard electrospinning [28].

Centrifugally spun polymeric fiber can provide ideal characteristics of tissue scaffolds. It should be taken into account that the appropriate scaffold needs to accommodate several competing needs: (i) a deterioration rate that accurately reflects the pace of regrowth of desirable living tissue, (ii) suitable layers and configurations of porous structure which permit cellular proliferation [26,29], (iii) adequate surface-volume ratio and a wide range of compositions of surfaces that promote cellular attachment, development [30], mobility, and differentiation [1,22,26]. Using various approaches, it is possible to develop templates for fabricating scaffolds with biocompatible and biodegradable properties [28,31]. Therefore, it is crucial to have an understanding of the effects of morphological alterations of the fiber matrix on enhancing bioactivity and controlled tissue development [21,32]. Introducing magnetic materials [33], hydrogels [34], nanoparticles [19,35,36], biomolecules [37], etc., may provide extended properties to establish cell frameworks for tissue regeneration. The centrifugal spinning process may be modified to produce antimicrobial fibers for wound dressings and other tissue-repair therapies [13,38,39,40]. Due to the presence of large pore structures in the fibers compared to the fibers made from other systems, it is possible to mimic the microenvironment necessary to mimic the extracellular matrix (ECM) [41]. Centrifugally spun fiber may provide closely matching mechanical characteristics, in addition to addressing some microstructures such as tendons, ligaments, ventricles etc., for tissue regeneration application [21,42].

Some excellent reviews have shed light on the many facets of the centrifugal spinning technique [3,43,44,45,46,47]. Even though there were a few reviews on centrifugal spinning that covered a wide range of topics in the biomedical industry, we were unable to find one that compiled all the available data on tissue engineering in one spot. In this article, we take a critical look at the latest developments in tissue scaffolds, artificial ECM, tissue regenerating fiber microenvironment, and cosmetics using centrifugally spun nanofibers made from synthetic, biodegradable, hybrid, and copolymer polymers. We also discuss the difficulties encountered during centrifugal spinning, its limitations, and potential future directions in expanding the technique’s utility for fabricating fibers for tissue engineering. Finally, some brief information is given on how to go about finding new polymeric materials with desirable multifunctional features and how to adapt machinery to generate fibers with unique morphologies.

## 2. Mechanism of Centrifugal Spinning

**Figure 1:**
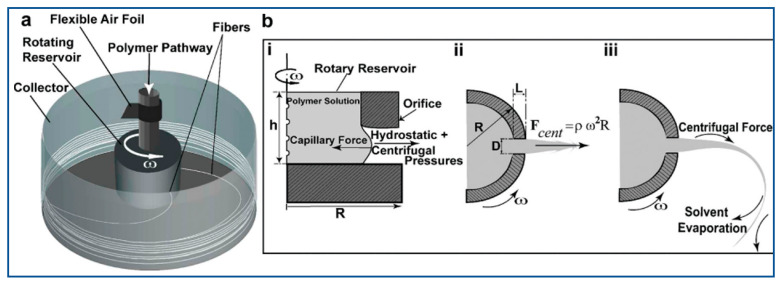
(**a**) A centrifugal spinning device constructed out of a hollow reservoir (**b**) mechanism of formation of nanoscale fiber through centrifugal spinning, polymer jet (i) initiation, (ii) stretching and (iii) solvent evaporation. Reprinted with permission from Ref. [28]. Copyright 2010, American Chemical Society.

A graphical interpretation of centrifugal spinning of polymer nanofibers is shown in Figure 1. The general setup for centrifugal spinning equipment, called a Cyclone machine, has been discussed in several articles [24,48,49]. An electrical rotor is coupled to a driveshaft where a spinneret is installed. Fibers are spun by forcing the substance into a spinneret’s precision nozzles via circular motion. The collecting process delivers the versatility to gather fibers either as a self-standing mat or as a continuous mat. To set down fibers on a platform or to process three-dimensional configurations, spinneret layout, angular or rotational speed, and enclosure aerodynamics are all crucial processing factors in centrifugal spinning.

This is an innovative technique that uses forces associated with centrifugation to mass-produce nano or microfibers. Even though centrifugal force spinning is based on the same mechanism as a cotton candy maker, there have been very few publications proving its efficacy as a nanofiber production method [23]. Polymer solution jets can be drawn from the spinneret without an electric potential difference between the nozzle and collectors; thus, no limitations are placed on using materials with low dielectric constants [15]. By ejecting fibers radially outward onto a collector, this technique is proven experimentally to produce nanofibers with a diameter in the nanometer range. When producing nanofibers by centrifugal spinning, it is required that the polymer is in liquid form. This can be obtained by dissolving the polymer into various solvents or heating the polymer to a temperature where polymer chains start to flow [49]. The polymer is loaded into a special container that rotates the fluid, which is then expelled as nanofibers onto a collector, maintaining a certain gap from the center [48]. In contrast, one form of spinning technique is the centrifugal electrospinning system, which employs a stationary spinneret. The static spinneret is wired to a DC power source, and the polymer solution flow is driven by external pressure. In this configuration, the produced fibers are deposited on a rotating collector [29]. In this approach, fiber alignment is dependent on the intensity of the electric field generated by the charge buildup on the deposited fiber [50].

For centrifugal melt spinning technique, both upper and bottom heater rings must be engaged to melt the polymer [16]. With no need for solvents, this method reduces processing time significantly. Polymers’ thermal properties, however, need to be considered in order to obtain useful design parameters. For example, the polymer must withstand extrusion temperature and should exhibit no crosslinking [51].

When the container with nozzles or holes is spun at an angle, a polymer jet is expelled from the opening and thins as it spirals outwards; eventually, the formation of a jet with a curved centerline happens [52] before reaching the collector. In the context of surface instability, the polymer jet formation instead of droplets is caused due to the viscoelasticity of the polymer solution [9]. Fiber production occurs as a result of distortion, elongation, and realignment of the polymer backbone as a result of a high-pressure loss at the orifice. The subsequent intense extensional and torsional loads are experienced by the departing polymer stream [24]. The imbalance among centrifugal force, viscous force & surface force can induce the jet to be broken up [53]. However, extended needle during rotation can lead to turbulent airflow, which in turn can lead to premature jet breaking [54]. An important factor is the polymer’s characteristics in its liquid or molten state by which the resulting fiber diameter and morphological features can be determined.

Badrossamay et al. demonstrated rotary jet-spinning (RJS), where the platform consisted of a motor shaft coupled to a solution storage container with two holes in the side walls for regulating flow [28]. A flexible airfoil is put on the shaft over the pool to aid in the collecting of fibers. Solvent evaporation can be affected by air turbulence created by an airfoil placed below the spinneret [6]. The reservoir is constantly replenished with polymer solution at a rate high enough to provide steady hydrostatic pressure and flow. A dual chamber reservoir (DCR), for example, is a feature in the RJS system in controlling mechanical properties, well fiber dispersion & spinning polymer blends in the same nano fabric [55]. The yielded fibers are collected on a steady circular collector or coverslips displayed against the collector’s side. The jet formation procedure primarily consists of several steps. First, a polymer jet is generated when the stream of precursor solution through an aperture is accelerated, (ii) as the jet widens, the surface area of the accelerated polymer flow grows, and (iii) solvent evaporates, which solidifies and shrinks the polymer jet. These steps are also mentioned in other sources as well [56]. Chen et al. outlined five stages of jet formation while describing the spatial movement of polymer solution [57].

At first, to drive the polymer fluid through the aperture capillary as a stream, the far end of the tubular is subjected to a combo of hydrostatic pressure and centrifugal pressure that is greater than the flow-resistant capillary forces. The polymer jet is stretched by the radial centrifugal force as it is directed toward the collection wall, yet its path is curved because of rotational inertia. In order to decrease the diameter of the emitted polymer jet from the nozzle to the collector, the jet must be stretched. Simultaneously, the polymer solution’s solvent dries, causing the jet to harden and constrict.

## 3. Factors Affecting Nanofiber Preparation

### 3.1. Polymer Melt or Solution Parameters

#### 3.1.1. Concentration

**Figure 2:**
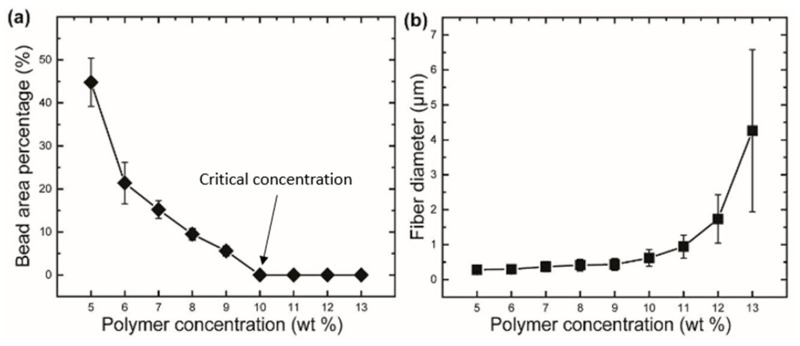
Variability of (**a**) beaded zone % and (**b**) diameters of recycled poly(ethylene terephthalate) (rPET) fibers fabricated from different polymer concentrations [62].

Properties of the fiber are more strongly influenced by polymer content, such as fiber diameter, bead formation, fiber alignment, etc. Increasing the amount of polymer in a solution causes fibers to develop with larger diameters [23]. The formation of fiber that is impervious to beading during centrifugal spinning requires a certain range of solution concentration [6]. While bead creation occurs at low polymer concentrations, increasing the concentration initially results in a combination of beads and fiber, and subsequently, beyond the critical concentration, continuous fiber development occurs given adequate chain entanglement [28]. The more polymer concentration appears, the more spanning of polymer chains shows sufficient entanglement of the networks [9] introducing a viscoelastic effect [58]. Chitosan/Polyamide (CP) viscoelastic properties are defined by adding chitosan to a polyamide solution [18]. Critical concentration refers to the concentration at which the fiber begins to exhibit homogeneous, continuous, bead-free fiber. By raising polymer concentration, the existence of beads along the fibers may be minimized [59]. The optimal concentration is the one that gives rise to the development of bead-free fiber with the most homogeneous morphology. Beaded fiber can be reformed at concentrations higher than the optimum. In particular, when PVP concentration is raised from 16 to 18 percent, such behaviors become apparent [56]. The optimal solution for spinning cellulose-based polymer fiber has a weight-to-volume ratio of 46–50 percent [39]. In other circumstances, the nozzle becomes clogged by the high viscosity, and even at a greater concentration, no fiber is formed [6]. The self-cleaning behavior of channeled nozzles with geometries should be addressed to prevent such blockage [10]. Very low concentrations are necessary for the essential polymer chain entanglement that promotes fiber production [39]. The entanglement concentration (C_e_) can be determined from the data of specific viscosity to understand the correlation between C_e_ and centrifugal spinnability. The highly branched structure of amylopectin provides an explanation for the diminishing entanglement behavior of starches, which is dependent on the amylopectin amount. After the concentration of the spinning fluid reaches a threshold, fiber production is achievable [60]. Fiber diameter can be lessened if the critical concentration is exceeded by adding an ionic compound, such as sodium chloride (NaCl) [61]. Figure 2 shows that there is a direct correlation between the fiber diameter and the concentration. For a given rotational speed, rPET fiber does not form a bead at concentrations higher than 10 weight percent. The C_e_ can be outlined by the critical chain overlap concentration (C*) [62].

For polymer gel, lower adhesiveness is required to overcome the attractive force between gel and spinneret material. The adhesiveness calculation helps detect spinneret concentrations of cellulose gel of different concentrations [63]. Increasing solvent volatility also leads to increased polymer concentration [64]. Amorphous ring or discrete diffraction caused by the alignment of polymer chains can be controlled by polymer concentration which is attributed to increased crystalline morphology resulting brittleness of nanofiber [20]. Compared to inorganic piezoelectric ceramics, co-polymer can be suitable for brittleness [55]. Surfaces with varying degrees of porosity, smoothness, wrinkles, and an intermediate condition between the two, known as grooves, may be produced by adjusting the concentration [6].

The solution parameters dependent on shrinking of nanofibers, such as polyethylene oxide (PEO) fiber trajectories, provide an inverse link between jet velocity and concentration [52]. Despite this, the changes in concentration have a far larger influence on viscosity than surface tension [9]. However, varying solvent properties can result in varying solvent evaporation rates, even when polymer concentrations remain constant [65].

#### 3.1.2. Molecular Weight

The continuity of fiber morphology is also dependent on polymer molecular weight (MW) which facilitates polymer chain entanglement. Critical chain overlap determined by the minimum molecular weight shows an impact on the fiber morphology [9]. It can be found that the polymer MW has inversed effects on the concentration range optimal for whirling cycles. The MW and the polydispersity degree are necessary to explain the complex interplay among the other parameters involved in morphology examination [10]. MW also favors the spinnability of the polymer blend. For example, higher MW is required to avoid droplet formation. Otherwise, a higher concentration is necessary to compensate for the influence of low MW of polymer. It is worth noting that when the MW is raised, the inherent viscosity rose alongside it until a threshold viscosity was achieved, at which point the fibers began to change shape from round to flat by increasing both their diameter and their inter-fiber spacing [11,12].

There is a need for further consideration regarding melt spinning. For instance, the polydispersity of thermoplastic polymers used in melt spinning should be low to guarantee a steady melt flow. Also, MW loss of polymer can occur considerably at the presence of moisture due to the hydrolysis of melt [51].

#### 3.1.3. Viscosity

A viscous polymer solution is required to create nanofibers with the desired spinning characteristics. Beyond an ideal range, the greater solution viscosity restricts solvent evaporation and necking, causing fibers to become thicker [28]. It is possible to use the viscosity of the solution as a surrogate for the degree of entanglement between the polymer chains. The viscosity of a polymeric solution rises as the degree of polymer network entanglement rises [7]. In addition to limiting solvent evaporation and preventing jet splitting, stretching, and thinning, the extended stress relaxation period in very viscous solutions promotes large fiber diameters [6]. Microstructural characterization based on o-Ps lifetime values, as detailed by Sebe et al., is in accordance with the results of the polymer’s viscosity alterations [8]. They reported a high degree of viscosity in the hydrogels formed from a centrifugally spun polymer, leading to reduced halogen content in the final fiber, where the relationship between viscosity and polymer concentration is dictated by the power law [64]. In contrast to the viscosity of bio-hybrid polymer solution, which rose with ascending protein concentration, the viscosity of pure starches with varying amylose contents increased linearly with increasing solution concentration [21]. The viscosity found from some fibers has an inverse relation to mechanical properties like shear rates [60]. Excessive viscosity, which hinders dissolving, can be caused by a polymer’s long chain length [66]. But increasing concentration beyond a certain limit can cause the phase separation to drop the viscosity. The concentration above 20% cooled polymer solution of lignin/TPU can display the upshot [67]. The low viscosity can be attributed to a concentration lower than critical concentration resulting in low binding energy in polymer chain. The intrinsic viscosity can be determined by using the Huggins and Kraemer equations [62]. The solution jet’s continuous structure is retained by its inherent viscosity [68].

For the melt spinning technique, it is possible to observe additional phenomena that affect viscosity. For instance, with the fast crystallization kinetics of polymers such as polybutylene terephthalate (PBT), the discrepancy between the processing and crystallization temperature may seem to have a greater impact than viscosity on the fiber diameter distribution [49]. In addition, viscosity that is too high can hinder processability. To ensure constant flow, the processing temperature range should be close to the decomposition temperature [51]. Increasing the temperature may accelerate the solvent’s evaporation but can further reduce the liquid’s viscosity [23]. The evolution of fiber shape may be studied better using various viscosity measurement data for melt-spun fiber. There appears to be a requirement for a greater quantity of fiber to be generated over a wider temperature range in order to overcome viscosity. Large, fractured, or necking-type behavior in both thick and thin fiber can occur in the higher viscosity PCL solution [22]. The lower the viscosity, the faster the jet, since the shear resistance of the polymer flow through the nozzle is reduced [52]. Using shear rate as a parameter in dynamic shear tests can decrease shear viscosity at a given concentration [23]. Apart from these phenomena, variations in the diameter of the nano- or micro-fiber can be caused by a localized raise in viscosity that promotes fiber coalescence [10].

#### 3.1.4. Surface Tension

To construct nanofibers using the centrifugal method, the centrifugal force must surpass the surface tension force [15]. Bead formation may be significantly affected by adjusting the surface tension of the polymer solution. When a polymer jet coming from the orifice is stretched, the surface tension of the solution can cause the polymer jets to break to produce droplets [69,70] or instability of the jet continuity [56]. Bead formation occurs since surface tension is decreased in spherical geometries [64], which may be compensated for by adjusting polymer concentration [28]. Nanofiber from starch also shows such surface tension induced by Rayleigh–Taylor instability to form beads [60]. The addition of surfactants such as sodium chloride (NaCl) to PAN fibers can reduce its surface tension resulting in significant decrease in fiber diameter. Comparing the composition of nitrogen and the nitrogen-to-carbon ratio of various samples’ surfaces shows that the proportion of nitrogen decreases with increasing salt concentration [61]. In addition, salt’s potential to stimulate the formation of thin veils in polymer solutions is an important consideration [71]. The fibers in a jet are aligned randomly due to the fluctuation in surface tension. Enhancing the intermolecular interaction between the links by adding gelatin to PCL/gelatin blends limits the jet elongation of precursor solution ejected from the spinneret, making the mixture more useful for cell culture research [72].

### 3.2. Machine Parameters

To produce fibers with small diameters, narrow distribution, and without any bead, it is necessary to acknowledge additional factors associated with cyclone machines such as angular velocity, solution outflow rate, duration of rotation, and nozzle size and geometry. Polymers have viscoelastic characteristics which allow them to exhibit stretching at high temperatures. The degree of the stretching can be correlated with their relaxation times. The solvent evaporation rate is also significant since it affects the viscosity and flexibility of the polymer. For modeling fiber production with the calculation of trajectory and ultimate diameter size, it is also necessary to have the diameter of the fiber-collecting curved surface and the needle orifice diameter [15]. Viscoelasticity is a time-dependent polymer property influenced by exterior force and time constant. The smaller/larger time constant shows the more elastic or viscous response of the polymer chains respectively [9].

#### 3.2.1. Flow Rate and Rotational Speed

The flow rate of the polymer melts or polymer solution plays a primary role in producing uniform nanofiber formation. Pressure-induced circulation from the outward centrifugal force acting on the solution of the reservoir entry helps determine the solution flow rate through the orifice [23]. Since the pressure within the reservoir shifts slightly because of the amount of fluid inside, this is still an additional indicator of the fluid’s fill level caused by the material feeding [73]. The slower volumetric flow rate leads the solvent evaporation for a prolonged period during the spinning process [58]. Under these conditions of limited evaporation, the still-wet fibers may combine to form a thin film as layers accumulate on the collectors [74]. Furthermore, diameter, homogeneity, and other fiber parameters are all affected by the rate at which the polymer solution flows. While the polymer melt is being pumped through a nozzle at a higher flow rate, the average fiber diameter drops. This happens when the solvent evaporates quickly while spinning [16]. Depending on the polymer solution, the distance between the spinneret tip to the collector up to a certain point, there is not any substantial reduction in fiber diameter. An optimal distance of 29.6 cm is reported for PHBV nanofiber production [73]. The rotational forces should be sufficiently large to overcome the surface tension of the solution or melt to create a polymer jet. It is possible for the low rotational velocity of the spinneret to induce the jet to break apart and form beads due to the surface tension of the polymer solution [74].

Moreover, according to the data supplied by Golecki et al., greater rotational speeds are needed to generate defect-free fibers when using less volatile, aqueous solvents, whereas lower spinning speeds are necessary when utilizing highly volatile solvents to form continuous fibers [64]. For the formation of the spinning jet, the critical angular velocity was directly related to the concentration of the spinning liquid and inversely related to the needle diameter [17]. It can be observed that the degree of bead development has an inverse relationship with rotational speed [75].

#### 3.2.2. Types of Collectors

Generally, the fibers are collected on a set of column collectors [13], a vacuum collecting system [14], deep dish fiber collector with evenly spaced standing steel pillars [23,39,76] in a form of a web. Fibers can also be deposited on a polypropylene substrate [76] using an air-free box to produce nonwoven fibrous films [39]. Prior to the spinning process, it may be necessary to directly deposit fibers onto a metal substrate, such as titanium foil, which has been cut to the appropriate dimensions and attached to alumina collectors with polytetrafluoroethylene (PTFE) tape [77]. Additionally, fiber can be gathered with a spinning cylindrical collector driven by a reciprocating device [55]. The spinneret or revolving reservoir is another collection point for fibers. These nanofiber scaffolds are tightly packed and well aligned because of the tension provided by this reservoir. This results in an improvement of cellular orientation, self-organization and cell adhesion [21,27,72]. Fiber collection can also be controlled in terms of the direction it comes from. Potter et al. invented a hand-held, portable centrifugal spinning apparatus that can gather generated fiber in the perpendicular direction using a flow-induced method [78]. Jao et al. described a system in which a rotating head was combined with an automated track collector to collect fibers in an aligned fashion. In contrast to the nanofiber mesh ring typically accumulated on traditional stationary collectors, such a process yields a steady stream of individually aligned nanofibers [79].

#### 3.2.3. Nozzle-Collector Spacing and Nozzle Geometry

The spacing between the orifices and fiber collectors has an effect on the shape of the fibers. Changing this spacing changes the liquid jet’s flight duration, which in turn influences the rate of solvent evaporation [6]. When the needle-collector spacing is less than the critical distance, the ejected jets have little time to span out, which aids in the formation of porous fiber, whereas once the needle-collector spacing has been increased past the critical distance, the regular dense fiber is visible [11]. The collection distance influences the elongation period of a whirling projectile in the air. If the collecting range is insufficient, the rotating jet cannot be completely extended, resulting in nanofibers with a greater diameter. If the spacing of the nozzle to the orifice is too great, the rotating jet is unable to reach the collectors [17]. In some cases, even though a small difference among the working distances does not affect the fiber diameter, the short distance presents large size distribution with twisted, broken, and beady fiber [22].

**Figure 3:**
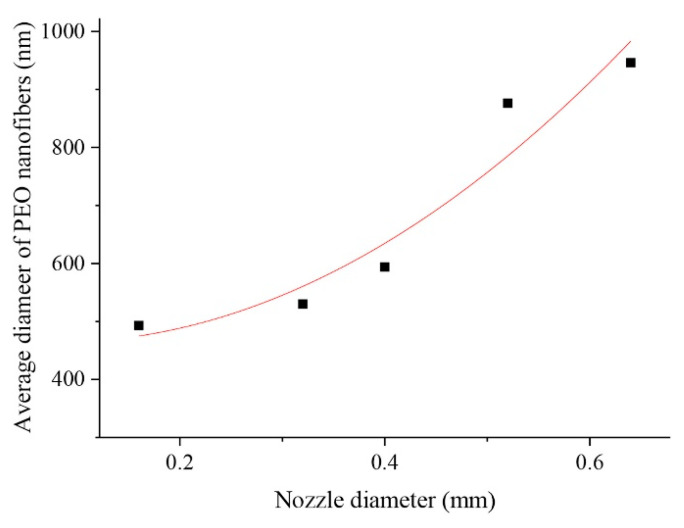
Effect of nozzle diameter on PEO nanofiber generated from 4 wt% PEO solution [17].

The configuration of the nozzle is a crucial factor in defining the diameter of the primary fiber and the course of the rotating or ejecting jet. The length of the nozzle can have an effect on the stability of jet continuity and stabilized flow. For polymers such as PEO, a nozzle that is 1.5 cm longer enables a more steady flow and enhances morphology as compared to a shorter nozzle. Additionally, the orifice diameter can be correlated to the fiber diameter, therefore a lower nozzle diameter in the millimeter range is necessary for nanofiber production [17]. Figure 3 depicts the change in diameter as a function of nozzle diameter. Reducing the length-to-diameter ratio of the orifice causes pressure loss at the orifice, increases the solution outflow rate, and decreases the rpm, resulting in fibers with a greater diameter [28].

#### 3.2.4. Air Foil

The quality of the air flowing through the system is a crucial factor that can improve both the fiber collection rate and the fiber characteristics. Incorporating an airfoil modifies the airflow distribution, which in turn modifies the fiber morphology via modulation of the solvent evaporation rate. Changes in fiber diameter and output rate are influenced by airfoil dimensions like length variation. The exclusion of an airfoil increases the likelihood that the collected fibers will contain some trace amount of solvent. This complicates both the process of collecting fiber and determining fiber production rate. According to studies, the longer air foil results in more effective fiber production. Additionally, the average fiber diameter is found to be smaller when using a longer airfoil (10 mm) in centrifugal spinning as opposed to a shorter airfoil (5 mm). This is due to the capillary thinning of liquid fiber jet prior to solvent evaporation from fiber exterior in large quantities [80].

### 3.3. Ambient Parameters

The rate at which the solvent evaporates from the polymer solution is dependent on its inherent volatility, which may be changed by environmental factors such as temperature and relative humidity, amongst others. When the solvent evaporates quickly, it speeds up the solidification process, which prevents the jets from spreading out as far. This occurs when solvents have a high degree of volatilization [28]. According to Shanmuganathan et al., the temperature is one of the parameters that has a greater influence on the features of the fiber when compared to another variable. The proportion of sub-micrometer fibers produced by the nozzle increased noticeably as a direct result of the temperature of the orifice being raised. In the process of melt spinning, increasing the temperature of the extruder enables the polymer jet to maintain its melted state for a greater amount of time, which promotes further elongation before the polymer crystallizes and becomes solid. It also shows that there is an inverse relationship between temperature and viscosity by demonstrating that there is a reduction of one order of magnitude in the shear viscosity of PBT polymer as the temperature increases [49]. The crystallization under molecular orientation inherits the process of structural changes and can be maximized by a strong dependency on temperature. Melt-spun fibers, such as polypropylene (PP) fiber, undergo rapid cooling near the nozzle’s exit due to the air stream generated by the spinning spinneret. This rapid cooling promotes the structure that covers a large area of crystals [16] and beads [22]. Although the pressured gyration process occurs at room temperature, the polymeric jet experiences a temperature shift due to the evaporation of the solvent. Solvent evaporation due to heat or frictional loss generated by high rpm will have an effect on the elongation viscosity, which is typically employed to assess the distribution and diameter of the fibers in the final product [9]. The temperature and humidity level of the area had been considered within 70–75 °C and 40–45%, respectively, to produce aluminum isopropoxide (AIP)/poly(vinylalcohol) (PVA) composite fibers [81]. Desiccating and storing fibers in a dry place is essential for maintaining a low relative humidity. When the relative humidity is low enough, pure, homogeneous, and disconnected fibers develop. Higher humidity levels impede fiber generation, which leads to thin film formation in the collector [71]. Therefore, spinning at lower humidity is required to develop uniform, continuous fiber. However, there are additional experiments conducted in more humid conditions that have been reported elsewhere [56].

## 4. Types of Materials Used for Centrifugal Spinning

Spinneret gauge shape, length from the collecting device to the orifice, rotating speed, and melt or solution concentration all have a role in determining the final web’s fiber production and shape during solution or melt spinning, respectively. Therefore, it is undoubtedly necessary to find the optimum rotational speed and solution concentration for successful fiber production which relies on the synergistic result of fiber output and fiber diameter. Varying the parameter can lead to better tunability of fiber morphology. Furthermore, it should be noted that beady morphology, low production of fiber, and discontinuity and nonuniformity of fiber must be overcome. Apart from common parameters, an additional parameter called working pressure involves when the technique is also assisted by solution blowing. The blend ratio of the polymer mixture should be considered to obtain the expected morphology. These factors have a significant impact on the various types of polymer fibers listed in Table 1.

## 5. Fiber Characteristics

### 5.1. Fiber Homogeneity and Bead Formation

It is quite difficult to achieve the appropriate properties without first obtaining homogeneous fibers. Adjusting the parameters above will yield the required morphologies, such as stable and uniform polymeric membranes [24,25], smooth, homogenous, continuous [18,19], multilevel structured [92] and long beadles [33,76] fibers. Fibers can create a spiral route by extending the collector distance, facilitating the extensional flow, and triggering evaporation to which evenly distributed continuous fibers can be generated [24]. According to Ren et al., the rheological characteristics of polymer liquid medium ascertain the final result. Morphologies, whether they are beadless or beaded fibers after the spinning [7]. The presence of beads can affect mechanical performance as well. Therefore, various process parameters must be altered in order to minimize beads. By varying these parameters, it shows that beaded fiber can be minimized by raising solvent volatility, viscosity, and rotational speed [64]. For a concentration of rPET fiber at higher viscosity, if the time scale of beading becomes longer than the time scale of solidification of polymer jet surface, it prevents Rayleigh instability resulting in bead-free fibers [62].

In some cases, under a concentration threshold, bead formation is inevitable. For example, in any concentration less than 9 wt% for polymethylmethacrylate (PMMA) [93], the beading morphology cannot be avoided. Surface tension applied force, and viscoelastic forces contribute to the creation of PVB, which can occur at concentrations less than 12 wt% [23]. With the increase of polymer such as PVDF concentration, the fiber diameter also increases with a reduction in bead formation, which yields continuous homogeneity along the fiber length [94]. This is why the critical entanglement concentration (C_e_) must be exceeded to form defect-free fiber and enhance mechanical properties [62]. Therefore, it is necessary to determine a qualitative measurement of bead percentage. Bead area percentage (BAP) can be measured to analyze fiber morphology or to investigate Rayleigh instability from following equation:

BAP = S_1_/S_2_ × 100%

where S_1_ and S_2_ are the total areas of beads in the measured SEM image and the area of the SEM image, respectively.

**Figure 4:**
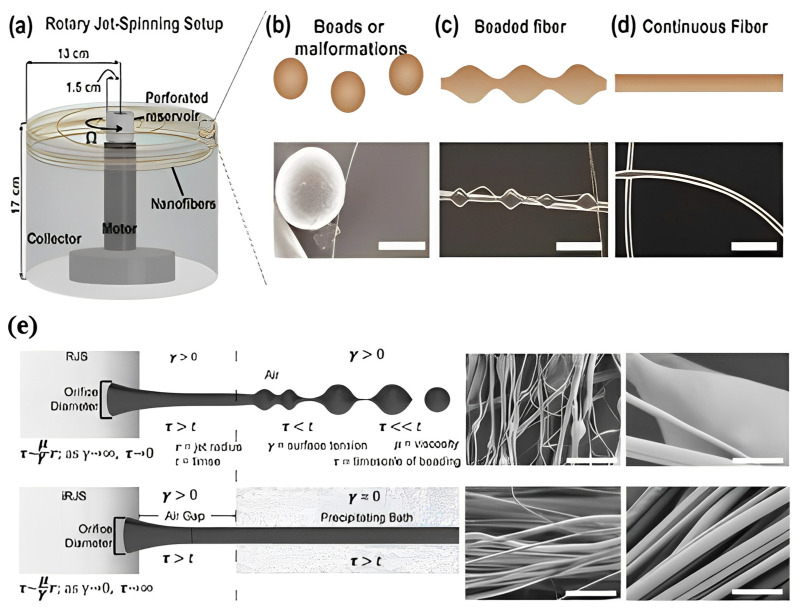
Variable PLA fiber morphologies. (**a**) Schematic of the spinning setup. (**b**) beads, (**c**) beads-on-string, (**d**) fiber showing continuity. Reprinted (adapted) with permission from [64]. Copyright 2014 American Chemical Society, (**e**) conventional nanofibrous spinning techniques that depend on volatile solvents result in beads. Fibers spun with the centrifugal spinning technique reduce surface tension due to the precipitating bath, stalling Raleigh–Plateau instability to generate bead-free fibers [20].

**Figure 5:**
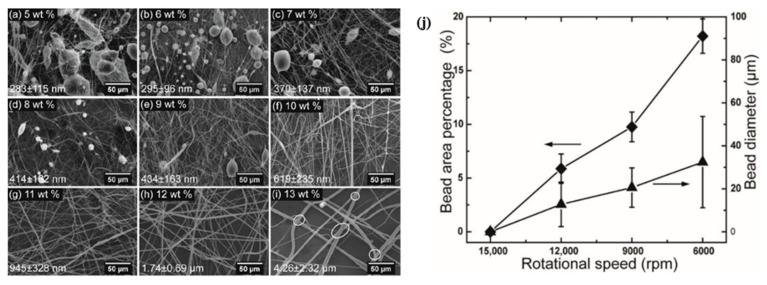
(**a**–**i**) SEM images of rPET fiber produced from various polymer concentrations, (**j**) percent bead area and bead diameter of rPET mats fabricated under different rotational speeds [62].

As stated earlier, if solutions have low viscosity and inadequate elastic contribution, the nanofibers start to break, inhibiting the development of homogenous fibers, and spatters or necklace-like formations known as “beads-on-string” are generated. The process of bead production and its other characteristics are also depicted in Figure 4. According to Figure 5, the beads-on-string fibers could be noticed when the rotational speed for rPET is decreased from 15,000 rpm to 12,000 rpm or below [62]. Furthermore, such morphology can also be obtained for polyamide 6 fiber at 15 wt% and 25 wt% concentration [95]. Under identical conditions, where fiber formation is possible, the diameter of the nozzle’s needle gauge may play a determining role in the existence of this structure. When viscosity increases, the viscoelastic jet takes longer to break up or does not break up at all, and beadless continuous long fibers are produced if extensional deformation owing to centrifugal forces is faster than the inverse of the fluid relaxation period. Bead-free continuous fibers are difficult to produce at temperatures when the temperature is inversely related to viscosity and directly proportional to the rate of solvent evaporation.

When phase separation suppresses the hydrodynamic instabilities, the timeframe of fiber beading is constrained by $\tau =\frac{µ}{\Upsilon }r,$ where μ is the solvent viscosity, *γ* is the surface tension, and *r* is the jet radius. During the solution spinning, if the polymer is miscible with the carrier solvent but the polymer still deposits, the surface tension of the polymer jet surface approaches zero, therefore, it increases the timescale of bead formation. Furthermore, fibers become bead-free, provided that the air gap is adequately small, such that the polymer solution attains the precipitating bath before beading occurs [20]. Golecki et al. provide a time scale for the solidification of the surface layer as it relates to the evaporation of the solvent to create a bead-free, continuously stranded fiber [64]. Furthermore, a technique of blown-centrifugal spinning can increase the rate of solidification by inducing airflow that inhibits bead formation [96].

The incorporation of nano filler into a polymer solution might alter the solution’s concentration, viscosity, or other characteristics, which can have little or no influence on fiber homogeneity. For instance, silver (Ag) nanoparticles in CA spun fiber do not affect the homogeneity; furthermore, the pure CA spun fiber has beadless and continuous morphology [39]. While the presence of bead formation is often seen adversely, there are certain novel microstructures that make excellent use of microbeads in the biomedical field [31].

### 5.2. Fiber Alignment

Fiber alignment is a necessary criterion to develop biocompatible composite material which can mimic skin, valve tissues, etc. Fiber anisotropy can favor cell alignment and initiate cell growth along the fiber axis [27]. For this circumstance, an understanding of what influences the anisotropy, and alignments of fibers are crucial.

The polymer jet trajectory is in a very complex 3D ‘‘whipping’’ way caused by bending instability rather than in a straight line. Aligned fiber occurs when most fluid jets elongate parallel to one another throughout the centrifugal spinning process. Filler such as carbon nanotubes (CNTs) along with the polymer can travel through the nozzles, therefore, the orifice can promote alignment of the polymeric chains as well [15]. From Gonzalez et al., the highly aligned anisotropic thin film can be obtained by using a rotating drum collector [97]. The orientation order parameter (OOP)-based characterization is also done for the fiber alignment reported by Badrossamay et al. [21]. It portrays an inverse correlation between fiber diameter and fiber alignment. In uniaxial systems, the parameter value is zero, whereas, in properly aligned systems, it is one. The increment of protein content in protein-polymer fiber can decrease OOP value owing to the existence of finer fibers resulting in less perfectly aligned fiber. FTIR achieved ratio between amorphous to the crystalline peak can yield a value that can be compared with respect to zero. The value represents the parallelism of the polymer chain to the nanofiber longitudinal axis and the well-integrated polymers in a multi-material sheet [55]. Due to the chain alignment along the fiber axis during fiber production, a substantial amount of pulling force at high rpm may occur on fiber filaments [73]. Ordered fibers may be more biocompatible because they promote cell contacts in an axial orientation [26]. Although it is crucial to get improved fiber alignment through fast spinning velocity, the melt-spun fiber may provide desired morphology with a better result of alignment [22]. For instance, highly aligned PCL implementation to produce reinforced hydrogel is an excellent method for avoiding excessive stiffness compared to tissues, organs, etc. [34].

### 5.3. Fiber Diameter Control

It is essential to regulate fiber diameter to produce mechanical properties similar to those of human internal organs, such as the fibrosa of the natural aortic valve [34], tendon, cartilage [27], etc. Many parameters such as solution concentration, composition, the spinneret rotational speed (rpm) [81], the air-to-polymer mass ratio [16], spinneret needle-to-fiber collector distance [77], nozzle diameter [6,98], spinneret diameter [99], blend ratio of polymers [36,67] have an obvious impact on fiber diameter and morphology [23]. Predicting the effects of variables on fiber diameter can be obtained using a quadratic model validated by residual plot [81]. The degree of fiber diameter distribution is dependent on spinneret speed. Faster rotational speeds and durations result in a smaller fiber diameter, a greater alignment of polymer chains, and the formation of an oriented mesophase characterized by rapid stretching and imperfect crystallization due to rapid solvent evaporation [75]. The fiber’s distance from the collecting substrate does not vary noticeably as the orbit progresses because of the constant stretching that occurs throughout the path. A significant shrinking of fiber diameter occurs at this time [100]. Sometimes a rougher surface is introduced when the fiber’s diameter is reduced [15]. Increasing fiber diameter causes a reduction of the acting centrifugal force on the polymer chain leading to an increase in the amount of mesomorphic phase, and thus to a decrease in crystallinity. This enables greater time for the crystallization process as opposed to polymer chain orientation which can relate to a high degree of crystallinity due to increasing rotational speed [101] and is associated with the amount of chain scission [29]. In this circumstance, a high rpm facilitates a large diameter dispersion, which may reduce the cooling rate. However, this is not always the case due to the inverse correlation between crystallinity and rotating speed and the fast rate of cooling [22]. Shorter working distance might play another role for high level or crystallinity.

In general, the fiber jet is supposed to be cooled by the traveling time from the spinneret tip to a collector when optimal distance is taken into account. In the shorter distance, some cooling takes place after the fiber reaches the collector allowing more crystallization. Larger fiber diameter is a direct result of increasing polymer content [56]. Thinner fibers obtained at lower polymer concentration could be due to lower viscosity or additional stretching before sufficient evaporation takes place for the onset of nanofiber formation [23]. The increase of PMMA concentration from 9 wt% to 12 wt% [52,93], PEO from 6 wt% to 10 wt% [52] leads to increase in average diameter by more than two-fold. In hybrid protein–polymer, increasing protein concentration promotes fiber diameter increase [21]. Higher spinning speeds often yield smaller output diameters due to reduced bead production [85].

The rotational speed is varied in order to optimize fiber diameter and uniformity. When the spinneret angular velocity was increased, the jet trajectories extended further outward making the fiber elongated [102]; evidence from this study shows that as spinneret rotational velocity rises, so does the throughput of the polymeric fiber jet. An SEM micrograph of the PEO and BEH-PPV nanofibers showed they have an average diameter of 300 nm produced by rotational speeds of the range of 3000 to 5000 rpm and diameter of 570 nm at a speed of 4000 rpm, respectively [15]. The reduction of fiber diameter occurs due to the inertial drag between the fiber and the atmosphere as the polymer solution jet dries [23]. The higher rotation speed means higher centrifugal force which induces the polymer jet extension and thinning which results in thinner fiber diameters [6,28]. But it is also possible to have reversed effects due to the higher rpm. As the rotational speed influence, the air flow, the solvent evaporation rate can be accelerated suppressing the polymer jet elongation resulting in thicker fiber [67].

According to the research of Golecki et al., fiber diameter grows as solvent volatility rises [64]. These findings imply that the drying process produces larger fiber diameters due to a higher polymer concentration and viscosity as a result of quicker solvent evaporation while using a higher volatile solvent. In this situation, the fibers harden rapidly before the polymer jet is stretched significantly, limiting fiber thinning. Amylopectin-rich starch-based fiber has been spun at 3000 rpm to form fiber with a diameter range from 0.75 to 2.25 µm indicating a narrow distribution [60]. The increment of rPET fiber diameter can also be attributed to the increase in the solution viscosity, which prevents the elongation of the polymer jet upon spinning [62].

Varying nozzle diameter can result in different mass throughput, which can either accelerate/decelerate fiber diameter. Another parameter, the inner diameter of the needle can affect fiber diameter which can be attributed to the formation of entanglement resulting in the change of mechanical properties of fiber [62]. The greater the nozzle diameter, the more the fiber diameter, and vice versa [67]. The relationship between fiber diameter and the parameters can be provided by the following equation:

D = a/R_C_^3/2^n

where D represents fiber diameter, a is the nozzle diameter, R_c_ is the nozzle–collector distance, and n is the rotational speed [6]. The fiber diameters may be slowly increased by shortening the collector distance as they had a small amount of space to be taken before becoming stationary [58]. The distance may control the capillary thinning time of fiber—so thinner fiber results from a larger working distance [67]. The reduction of orifice diameter means a lower material flow rate. Due to this fact, a shifting of the fiber diameter distribution towards fiber with a smaller diameter is likely to occur [103]. Nanoparticle insertion can enhance polymer fiber’s multifunctional features such as cell adhesion, growth, etc., without affecting its diameter owing to electrostatic interactions [33].

### 5.4. Fiber Porosity

Analysis of porous structure, pore distribution, and the porous area is necessary to conserve a substantial amount of energy that might be utilized for stretching jets into smaller fibers, and perhaps for ion and nutrient exchange mechanisms in tissue interfaces. The porosities assess the scaffolds’ surface strength, permeation, and mechanical stability [72]. Centrifugal spinning can provide porous structures like TAF [24], PAN membrane [25], poly (D, L-lactic acid) (PDLLA) fibers [26], Janus-type polymer [27], etc. With this method, it is also possible to obtain a large number of micropores with bead-free features simultaneously [25]. A previous study suggests that a solid, homogeneous interface can possess uniformly distributed smooth and wrinkled layers on the porous surface of nonwoven fiber mats [71]. In the centrifugal spinning technique, when solvents evaporate slowly, a high porosity can emerge due to a process called vapor-induced phase separation [24,104,105]. Another factor for pore formation may be the vapor pressure difference between solvents [84]. The porous structure is commonly associated with a rough surface area, which may or may not have an effect on increased cell adhesion [26].

Hou et al. observed that in EC/PVP fiber formation, using the low ratio of ethanol/water with a high EC/PVP ratio can provide maximum porous feature [84]. The calcination process, which involves the removal of carbon material, increases the porosity inside fiber structures. When the calcination temperature is quite high, the size of the porous structure increases while the pore volume decreases, and also the surface area of the fibers is reduced [81]. Loss of binding polymers results in a porous structure, which may be seen when polymer blends are subjected to heat treatment. Micro-porous features and the aligned of polymer molecules can be influenced by the supramolecular structure of the initial gels in the polymer blend. It also has an effect on mechanical properties [63] such as degradation of mechanical strength by the removal of binding polymer leaving the open porous feature [66]. The cellulose–Ag composite membrane exhibits an interconnected microporous 3D network structure. Due to high porosity, they show a larger specific surface area.

**Figure 6:**
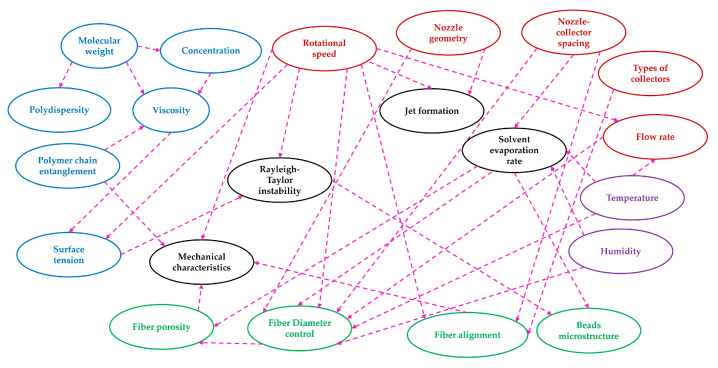
Schematic of the interdependence between the ambient parameters (purple), polymer melt or solution characteristics (blue), machine parameters (red), and other dependent variables (black) on the fiber morphological characteristics (green) derived from various sources [6,16,17,22,60,62,64,74].

The presence of cellulose leads the material to have highly inherent hydrophilic characteristics, which improves its ability to absorb and retain water [39]. The larger porous structure also contributes to the high cellular permeability of the ternary membrane which might have functional features with increased flexibility like skin cell flexibility [76], cell migration, and proliferation [34]. These porous characteristics allow for the passage of nutrients and gases between tissue and culture medium at various depths [26]. Also, pore formation is more pronounced in micro particle embedded composite than nanoparticle composite while both contribute to ion transportation [106]. Besides, large pore distribution, for instance, a pore area between 2 to 2561 μm^2^ is sufficient for cell migration [26]. The melt spun process allows for a porous structure to be generated without the need for embedding particles; these pores are then useful for promoting cellular infiltration and proliferation [29]. The optimal porosity for tissue scaffolds is sometimes considered between 60% and 90% [72]. In summary, it can be deduced that the factors that influence the fiber morphologies have a strong interdependency with one another, as shown in Figure 6.

## 6. Nanocomposite Application for Tissue Engineering

### 6.1. Scaffold Fabrication

#### 6.1.1. Tissue Template

Nanoscale fibrous scaffolds should exhibit higher surface area which can offer an ideal template for cells for seeding, migrating, growing, differentiating, etc. In addition, the pace at which such a structure degrades should be comparable to that at which real tissue regenerates [1]. For natural tissue regeneration to be effective, new fibrous structures must be developed with distinct designs that promote cell implantation and cell proliferation. The creation of reproducible and biocompatible three-dimensional scaffolds with large pore sizes produced by centrifugal spinning can extend the permeability and structural integrity of cell ingrowth compared to electrospinning resulting in bio-matrix composites for different tissue repairs and substitution processes. Development of such 3D scaffolds has been introduced for cell infiltration, migration, adhesion, and growth [30]. Cell adhesion is favored by fibers with smaller diameters when compared to large-sized fibers [33,86]. Recently, many investigations have been started to pay attention to making scaffolds with synthetic biopolymers or biodegradable polymer nanofibers. Biodegradable aliphatic polyester like polycaprolactone (PCL) [28], poly (D, L-lactic acid) (PDLLA) and animal-based gelatin [107] have been centrifugally spun to produce homogenous and sub-micrometer fiber for medical applications [75]. According to osteoblast cell viability tests, the PDLLA system can stimulate more robust cell responses [108]. Nanofibrous alginate scaffolds are biocompatible and low toxicity and they also possess reconfigurable size and mechanical characteristics making them potential candidates for skeletal muscle tissue engineering [20]. Alginate can be used with poly(lactic acid) (PLA) fiber to facilitate adhesion to the cellular matrix and prevent cell deformation, resulting in an increase in yield stress owing to pseudoelasticity. Due to the polymer’s gelation process and subsequent centrifugal spinning, alginate is uniformly distributed throughout the main polymer matrix. Therefore, alginate promotes the hydrophilicity of PLA, and therefore provides compatibility with the hydrophilic environment [31].

**Figure 7:**
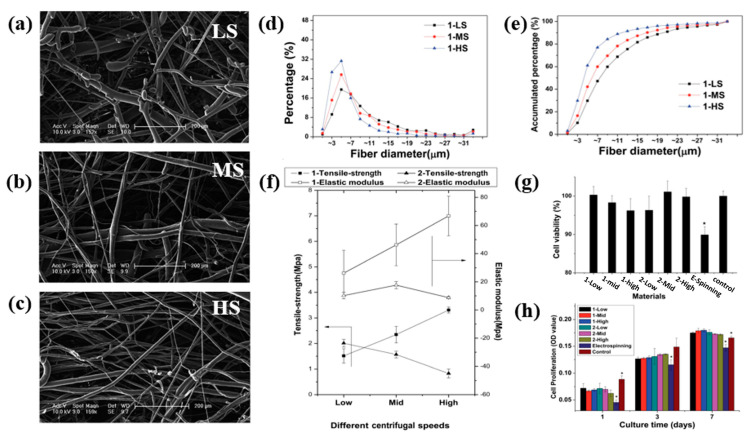
SEM image of melt−spun PLA fibers at various rotational motions (**a**) low speed (LS) (**b**) mid−speed (MS) (**c**) high speed (HS) (**d**) Frequency (**e**) accumulated frequency distribution of fiber diameters (**f**) effect of speeds on mechanical properties (**g**) cell cytotoxicity and (**h**) cell proliferation on the fibers. Asterisk (*) denotes statistical significance *p* < 0.05 [29].

Polymers have been processed using melt spinning, a solvent-free method of centrifugal spinning, to create biodegradable nanofiber for cell growth. The three-dimensional structure of polylactic acid (PLA) was examined by Zhou et al., who reported that it had a more porous structure, low cytotoxicity, and a wider diameter distribution. Because of their effect on cell interaction and proliferation, large diameter distribution paves the way to address a wide range of tissue engineering challenges such as permeability to facilitate ingrowth tissue. Based on the findings of this study, it is evident that the cells on this melt-spun fiber may expand and orient themselves considerably along the fine threads while bypassing the coarse fibers. Figure 7f further demonstrated that an increase in rotational speed resulted in enhanced mechanical properties [29]. The attachment of stem cells is affected not only by the diameter distribution but also by the magnitude of the diameter. An increase in fiber diameter, for instance, promotes stronger cell adherence to non-woven textiles, leading to elevated alkaline phosphatase activity and osteocalcin levels [32].

The bio-functional feature of anisotropic scaffold fibers spun by the centrifugal method has been proven by examining their capacity to promote cell development and differentiation using a range of cell species. Pure protein scaffolds sometimes cannot be easily fabricated due to their instability in an aqueous environment without glutaraldehyde fixation. It is plausible that the incorporation of protein into the PCL framework will affect fiber surface characteristics and secondary structure, as well as introduce binding domains for cell attachment, hence providing favorable features for cellular adhesion and proliferation. There have also been some investigations carried out on the neurite length and branching in the context of neural tissue engineering [21].

Scaffolds made from PCL-based spun fibers can also promote cell proliferation and differentiation [22]. In PLLA/PCL fiber as a scaffold for cell growth, it is necessary to achieve a small diameter suitable for the cell to adhere to the support structure [88]. In these cases, PCL can be compounded with other substances to produce a substance with enhanced properties such as cell adhesion, cell viability, immunostaining [34], proliferation, and neural differentiation [33], etc. The anisotropic networks contribute significantly to efficient neurological damage, making them an attractive target for an investigation into injectable forms of neural tissue regeneration. It is possible to create aligned hybrid alginate using PCL nanofibers that have been magneto-responsively spun using a centrifugal spinning technique. In addition to this, the incorporation of superparamagnetic iron oxide nanoparticles (SPIONs) into PCL enhances the activation of ion channels in intracellular signaling while retaining the material’s cytocompatibility and distinctive magnetic characteristics, and durable structural attributes. A magnetic field was applied externally to fine-tune the alignment of nanofibers due to the presence of SPIONs, resulting in their actual synchronization in the same direction [33]. Ravishankar et al. tried to replicate the physical properties of a heart valve whilst also keeping cell viability robust, demonstrating positive stimulation, and promoting tissue growth. Photocrosslinkable hydrogels such as gelatin, chondroitin sulfate (CS), hyaluronic acid (HA), etc., were used and reinforced with anisotropic PCL fiber to create a composite material called a fiber reinforcement hydrogel (HCs) [34]. The composite’s use of centrifugally spun fiber allows it to overcome the increased stiffness relative to native tissues that are caused by the absence of anisotropy in earlier studies [109,110]. Furthermore, the incorporation of hydrogels in fibers increased the permeability of the nanocomposite, therefore creating an optimal milieu for cell penetration and proliferation. Due to the presence of this hydrogel, cell adhesion and migration increase, hence enhancing the ability of valve interstitial cells (VICs) to secrete elastin [34].

**Figure 8:**
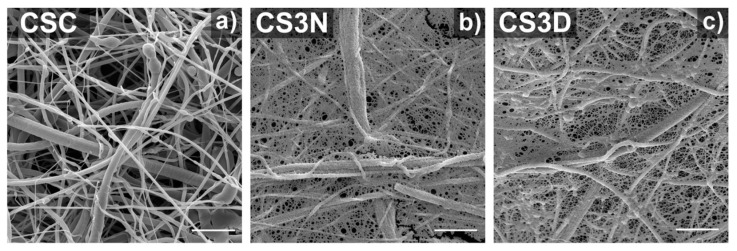
Centrifugally spun fibrillar scaffolds in (**a**) control, (**b**) differential, and (**c**) non-differential medium with adherent platelets were observed by scanning electron microscopy. Reprinted (adapted) with permission from [37]. Copyright 2014 Elsevier.

Polymeric fiber scaffolds can be designed with antimicrobial and bioactive fibrous elements to encourage cell framework contact during tissue regeneration as they possess similar characteristics to extracellular matrices with specific morphology. The PDLLA-based hybrid scaffold developed by Padilla-Gainza et al. exhibited minimal toxicity, high cell survival (over 80%), and favorable cell morphology during in vitro cell culture [26]. According to the findings of this investigation, the centrifugal spinning technique was used to evenly distribute the antimicrobial zinc oxide (ZnO) and bioactive hydroxyapatite (Hap) particles throughout the fiber system. The cells remained in close proximity to the fibers, and cytoplasmic extensions resembling filopodia were seen emanating from the cells and approaching the fibers. In terms of bacterial activities such as adhesion and growth, two reversed phenomena can happen. One, the presence of surface charge of the fiber materials can impact bacterial adhesion while causing inhibition to their growth [35]. Second, the polar group present in the Hap can promote bacterial growth and adhesion [111]. The characteristics provided by the enzymatic activity of alkaline phosphate and mineralization, ZnO incorporation may accelerate the regeneration of bone tissue [112]. Scaffolds that have been functionalized to serve certain purposes can greatly enhance the success of tissue regeneration. Cell metabolic activity and osteogenic differentiation were studied by Lukáová et al. using functionalized platelets on centrifugally spun poly-ε-caprolactone. The results of the study demonstrated that osteoinduction by platelets is enhanced in inorganic phosphate and ascorbate-rich settings. Platelets in the differentiating medium increase the metabolism rate by releasing biomolecules, which in turn stimulate cell proliferation. Platelets are present in the fibrillar scaffolds illustrated in Figure 8 micrographs [37].

Polymers with a hydrophilic character are preferred because of the improved contact they allow in the in-vivo microenvironment. Polyvinyl alcohol’s hydrophilicity renders it a possible option for use in the medical domain. To increase their processability for potential tissue engineering applications, Akhtar et al. studied PVA-based nanofibers with alginate di-aldehyde (ADA), ADA/gelatin (Gelatin), and other content to regulate the viscosity and degradation kinetics. Bioactive glass (BG) nanoparticles doped with copper and silver (Cu-Ag) and possessing a mesoporous structure were incorporated into the system via dip coating technique to enhance their antibacterial properties [36]. Other metallic contents such as Zn and Sr can be doped in the BG support for the regeneration of soft and hard tissue and possible enhancement of osteoconductive, angiogenesis of tissue scaffolds [19].

#### 6.1.2. Tissue-Wound Healing

**Figure 9:**
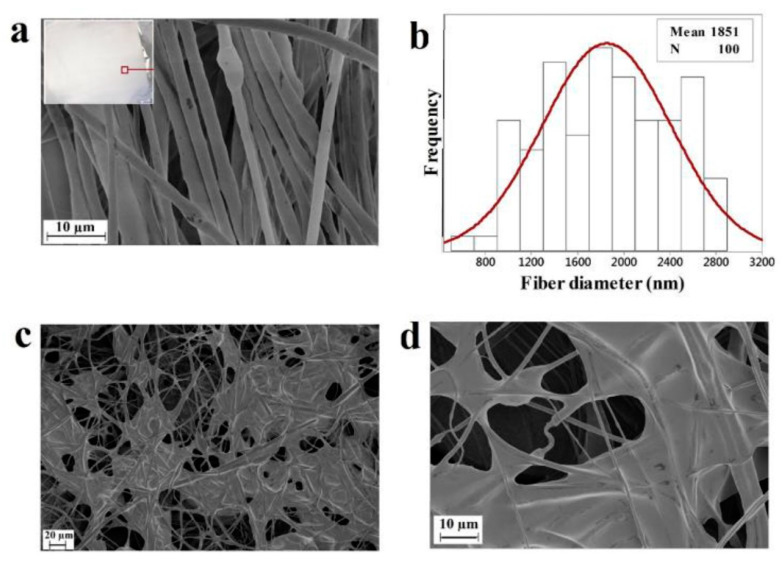
(**a**) Micrograph of polyhydroxy butyrate (PHB) fibers with fiber mat (inset image) and its (**b**) diameter distribution, and multiple magnifications of (**c**) 20 μm and (**d**) 10 μm of a PHB film that had been covered with SOJ [86].

Fabrication of biocompatible Polymer nanofiber can be implemented in human skin for wound healing. In dermal tissue repair, fibroblasts migrate from the surrounding healthy tissue into the fibrin clot, where they secrete collagen and several extracellular matrices (ECM) elements. The direction of the fiber matrix guides the fibroblast’s migration on the fibrin clot via contact guiding [72]. In comparison to randomly distributed fibers, ordered ultrafine fibers have the potential to be a more effective option for the promotion of cell growth and the regulation of cell migration [113]. With the centrifugal spinning technology, protein-based polymer nanofiber with a greater degree of alignment can be achieved for direction-assisted tissue regeneration. Also, fibers made from polycaprolactone (PCL) can play an important role in promoting wound healing. PCL/gel [72], and PCL/PVP [114] aligned ultrafine fibrous matrices can progressively generated by centrifugal spinning technology. Norzain et al. reported a triangular-prism-like micro-structured PCL nanofiber scaffold that could even attain 97 percent wound repair and tissue regeneration with complete epithelialization [115]. The micro-macro structural characterization of complex fiber mats like PVP/poly(vinylpyrrolidone-vinyl acetate)/iodine developed by the procedure facilitated the identification of the appropriate wound dressing composition [8]. Centrifugal spun samples can be seeded with valve interstitial cells (VICs) used to assess cell metabolic activity [21]. Reducing infection in wound healing and treatment, sour orange juice (SOJ) can be incorporated into polyhydroxy butyrate (PHB) fibers as an antibacterial agent with improved cell adhesion and proliferation. PHB fibers showed the effectiveness of trapping liquid agents into their system, providing a moist medium to promote healing, as illustrated in Figure 9 [86]. The PHB/SOJ scaffolds showed antibacterial activity of up to 152% and 71% after an antibacterial test conducted using the disk diffusion and dilution methods. Cell viability studies have been performed by examining fibers using MTT assay [38]. Disk diffusion method also applied on cellulose-based materials. The silver particle on fiber surface which actively interacts as nuclei sites with CA microstructure during spinning process plays a more vital role in antimicrobial activity [39].

The antibacterial activity can be tested via the cell counting method [76]. Cremar et al. demonstrated, using the cell-counting method, that the inhibition zone surrounding the fiber mats completely inhibits the development of gram-positive bacteria. In this work, TA inclusion is followed by alkaline-induced crosslinking to improve the structural integrity of fibers for use in wound dressings in various environments [13]. It is possible to build flexible Ag-based cellulose membranes that maintain conformal contact with uneven and complex wounds. They show a high water retention capacity, that may create a longer wet microenvironment on the wound, hence promoting re-epithelialization and reducing scab development [39]. Also, Ag-embedded PEO porous fiber may hinder bacterial action by disrupting bacterial structure [116]. The ideal wound dressing must absorb excess exudates without leaking to its surface; it must, in a sense, have water absorption properties [39,76] which promote an optimal environment of healing [87]. The obtained EC/PVP fiber from a higher ratio of water offers much surface area for efficient fluid absorption, cell attachment, and proliferation for wound healing [84].

**Figure 10:**
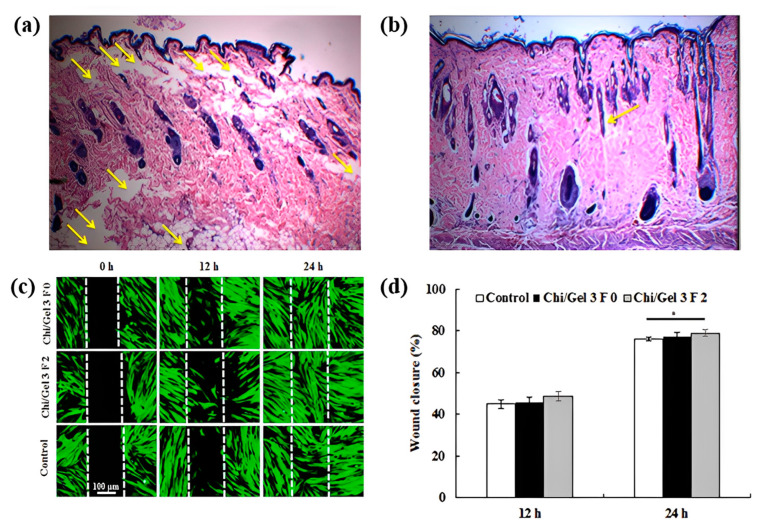
(**a**) Standard and (**b**) fiber-treated repaired tissue sections, (Arrows indicating sections stained with hematoxylin and eosin). Reprinted (adapted) with permission from [72]. Copyright 2014 Elsevier, (**c**) evaluation of in vitro wounds using the scratch assay test and contrast to a control condition, (**d**) quantitative investigation of cellular migration (* *p* < 0.05) [41].

Chitosan’s widespread popularity stems from the positive charge distribution and the bioactivities it possesses. Further, this polysaccharide’s configuration, which is identical to that of glycosaminoglycans, allows it to reduce the appearance of lesions on the skin. It does so by including glusamine and N-acetyl glucosamine. As can be seen in Figure 10, the chitosan-based fiber can also stimulate cellular migration [41]. It is commonly combined with other polymers to enhance their functionality. Due to the anti-bacterial activity of tannic acid (TA), chitosan/TA-based fiber can be processed while promoting activity by adding cinnamaldehyde (CA) and AgNPs. The growth inhibition zone is an observation to understand the activity against gram-positive and gram-negative bacteria, such as *E. Coli* found in the human intestine and nosocomial infections [39,76].

When crosslinking is applied to the healed collective tissues in the presence of a biocompatible crosslinking agent, such as CA, the nanofibers in the chitosan (CS)/PVA ternary composite become water resistant. The ternary fibers have enhanced mechanical properties suitable for skin functioning capacity with structural integrity [76]. Boyle et al. discovered that pullulan (PL) combined with chondroitin sulfate fiber has anti-inflammatory properties that reduce inflammatory chemokines that regulate irritation in wound infection [87]. Evaluation of dermal and epidermal layer recovery, as measured by epithelium permeability, depth, and scar size, is a critical factor in the healing process and subsequent tissue regeneration. Since this is the case, microscopic monitoring of these tissues over time is essential [117].

### 6.2. Artificial Extracellular Matrix

The dependence on medical treatment for the regeneration of tissues or organ become converged into fabricating scaffolds from natural and synthetic polymers using a variety of processing techniques. Mimicking collagen structures, referred to as extracellular matrix (ECM), can assist in duplicating intercellular reactions and promote the intracellular with an addition to the ideal biological environment. Embryologic development, organogenesis, cell proliferation, and wound regeneration. Cell viability studies obtained from Chitosan/composite fibers suggest its effectiveness in mimicking ECM of skin while protecting against bacterial infection [13].

**Figure 11:**
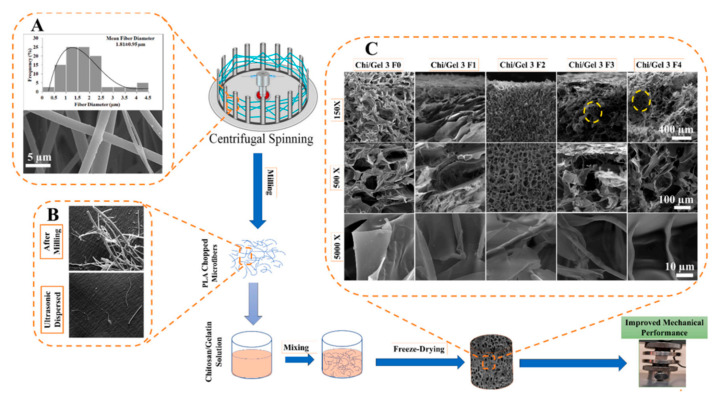
The fabrication of an artificial ECM scaffold, schematically. Centrifugally spun PLA fibers (**A**,**B**) and hydrogel scaffolds with fiber reinforcement (agglomeration of PLA-CFs is denoted by the yellow dotted line circles) (**C**). Reprinted (adapted) with permission from [41]. Copyright 2022 Elsevier.

The artificial ECM requires the characteristics of biocompatibility, permeability, and structural rigidity with the porous feature. Along with pore size and distribution, their connectivity also plays a critical role. To promote such characteristics blending the polymer with hydrogel can achieve controlled and regular pore morphology and higher water absorption. Chitosan/gelatin composites strengthened with centrifugally spun polylactic acid were developed by Eftekhari-pournigjeh et al. The freeze-drying and crosslinking procedures were carried out after the PLA fibers were milled and dispersed in the chitosan/gelatin blends. Increased contact surface for cellular matrix development is made possible by its high porosity (94–96%) and greater fiber presence. Figure 11 represents distinct porosity characteristics for various chi/gel contents. The study’s findings demonstrate that the hybrid can serve as an ideal medium for the preservation of keratocyte phenotype and the repair of the damaged corneal epithelium [41].

**Figure 12:**
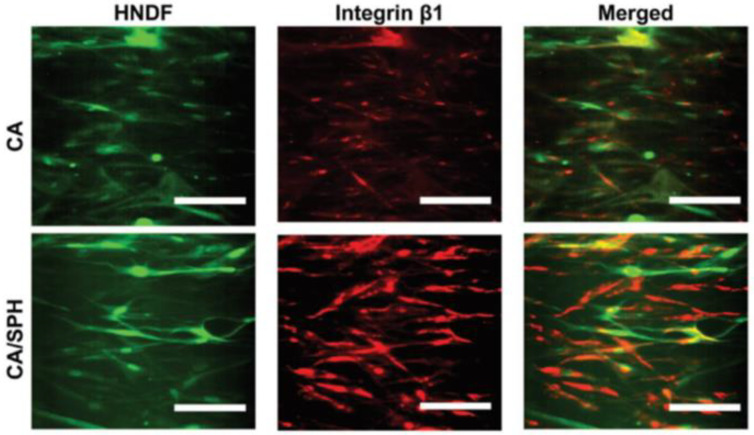
Expression of the integrin β1 by fibroblasts in vitro, as shown by immunostaining [117].

The bioactive compounds in protein-based artificial ECM may be analogous to those in natural ECM proteins and estrogen. Soy protein and its extract form nanofiber, which can restore the humid microclimate. Furthermore, its phytoestrogens can connect with estrogen receptors, influencing the growth of several body tissues. Fabrication of plant hybrid cellulose acetate (CA)/soy protein hydrolysate (SPH) using centrifugal co-spinning technique has been demonstrated to recreate the cutaneous milieu successfully and have a high-water retention capacity. By incorporating polar moieties like hydroxyl, amino, and carboxylic groups into the fiber, SPH increases hydrophilicity and, in turn, cell adhesion. Additionally, cell migration, fibroblast intrusions, and minimal cytotoxicity were observed after functionalizing CA nanofibers with SPH to improve surface roughness. Moreover, in vivo research has shown that upregulating integrin β1 expression aids in tissue repair, as depicted in Figure 12 [117].

### 6.3. 3D Cell Structure

**Figure 13:**
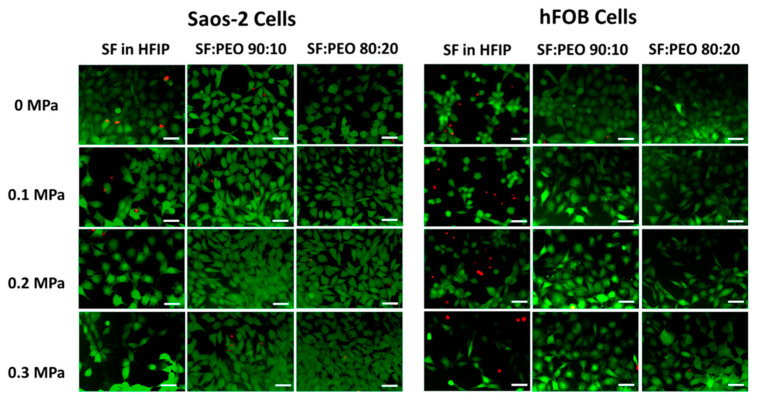
Cell viability experiment in SaOS-2 and hFob cells: typical fluorescence microscopy photos of information obtained from each specimen, with green and red fluorescence showing live dead cells, respectively. Reprinted (adapted) with permission from [89]. Copyright 2022 American Chemical Society.

It is possible to develop 3D cell culture models using the centrifugal jet spinning method in addition to 2D tissue scaffolds. By adjusting process parameters, including rotating speed and solution composition, Khang et al. synthesized Janus-type polymeric nanofiber with a biphasic feature [27]. A Janus-based polymer can produce a more extensive porosity characteristic and superior mechanical capabilities when combined with other biodegradable polymers, such as PCL with gelatin. The obtained fibers show comparable mechanical properties (tensile strength of ~10–13 MPa) with collagen fibers (~1–7 MPa) found in tendons, ligaments, and bone. Cell attachment, alignment, and elongation were shown to be positively affected by gelatin concentration, as indicated by alignment angle and aspect ratio. Improved mixing of the two phases (PCL–gelatin) is facilitated by increased rotational motion and solution viscosity, leading to less biphasic behavior. Badrossamay et al. also developed PCL/gelatin and PCL/collagen nanofibers with a higher degree of alignment for cell culture study [21]. Cell viability may be quantitatively evaluated with the MTT test, allowing for a qualitative biocompatibility evaluation. Research by Loordhuswamy et al. on a similar system demonstrated the efficacy of the matrices in vitro and in vivo environments [72]. Viable cells decompose the MTT reactant in the intracellular mitochondrion and create deep-colored formazan crystals that may be solubilized and used to determine the fraction of cell viability based on their maximum absorbance. Figure 13 illustrates cell viability evaluation by identifying living and dead cells [89]. Assessment of cell viability can be done quantitatively. The cell viability percentage (CVP) can be measured using the following equation:

CVP = A_s_/A_c_ × 100

where A_s_ and A_c_ represents the absorbance of the scaffold and control group, respectively. Changes in the absorbance of these scaffolds over time are evidence of cell development and replication [41]. Cell studies reveal suitable cell growth and attachment, while higher surface roughness of fiber mats improves cell adhesion and proliferation [86]. On the other hand, fine composite fiber or fiber surfaces without roughness can also function as noncytotoxic scaffolds for cell growth [13].

### 6.4. Cosmetics

Skincare masks such as topical creams, lotions, or ointments require hydrophobicity for being unaffected in humid weather when applied to human skin. Centrifugal melt spun copolymer fiber can be attempted as a waterproofing agent in cosmetics and sunscreen [90]. Sunscreens have intricate chemical compositions, mostly based on water in an oil emulsion, which contains a mixture of organic/inorganic and lipophilic/hydrophilic compounds [118]. For use as a skin care product, for instance, virgin coconut oil (VC) extracted from mature coconuts can be encapsulated in nanofiber [119]. However, the skin can become contaminated using commercialized items like lotions and films, and airflow might be obstructed. These drawbacks can be addressed by using a newly created fiber mask [40]. The hydrophilic nature of protein-based polymer coupled with the higher surface area can promote the absorption of humidity [120], and potential bioactivities [87]. TiO_2_ continuous fiber [121], nanocrystalline TiO_2_ fiber [122] prepared by centrifugal spinning can be widely used as the inorganic filter of sunscreen due to its effective UV reflection, absorption capacities, and adequate tolerance by human skin.

**Figure 14:**
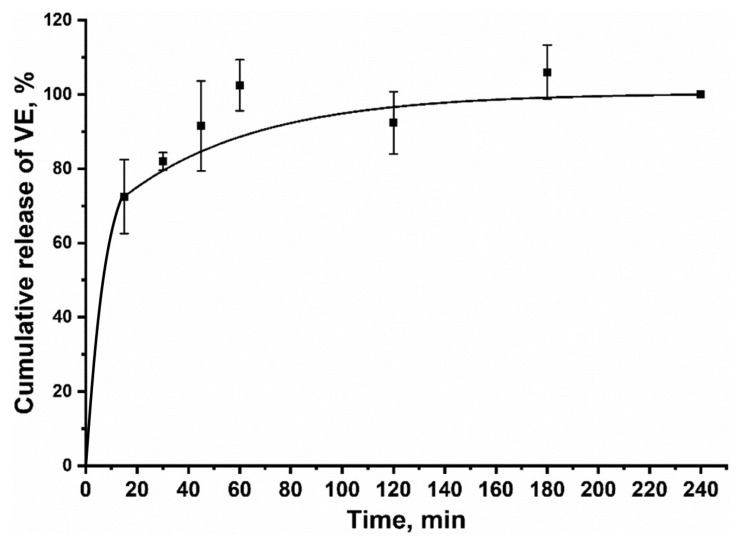
Vitamin E (VE) is released from fibers made of a polymer called GA: PEO, which contains the antioxidants vitamin C and vitamin E. First 15 min indicates a burst release (~72%) [40].

Vitamin addition to nanofiber materials is a promising skin tissue protection and regeneration technique. Rihova et al. used low molecular biopolymers such as gum materials with PEO nanofiber to study vitamin E release for cosmetic purposes. Gums and polyethylene oxide (PEO) were shown to have a weak secondary interaction, such as hydrogen bonding, which led to a decrease in fiber formation. The diameter of the fibers influences how quickly they discharge their adsorbed vitamin contents. In the absence of a non-polar solvent, where vitamin C would normally be dissolved, only vitamin E is released, perhaps because its oxidation is prevented by the presence of vitamin C, an antioxidant. Furthermore, vitamins’ prolonged release period makes them a viable active material for critically injured skin that demands prolonged topical therapy. Figure 14 exhibits the cumulative release of vitamins over time [40].

Cosmetics, lotion, and other soluble compounds all employ copolymers as water-resisting ingredients. One possible use for copolymers such as polyvinylpyrrolidone/1-triacontanol (PVP/TA) is as an adsorbent capture medium. Furthermore, they can also be employed to improve the efficacy of skin-protecting compounds with multiple uses. Due to triacontene’s inclusion, PVP’s hydrophilicity can be disguised by the formation of tiny lateral crystals surrounding the amorphous framework. Because hydrophobic behaviors with higher surface area boost adsorption properties, they may find use in the cosmetics industry, colorant binders, and other applications [90].

## 7. Challenges

Despite the progress, many essential matters are still required to focus on—for example, the homogeneous distribution of nanoparticles in the polymeric medium. The aggregated particles in the fiber system operate as stress concentrators and accelerate material failure if they are not disseminated uniformly throughout the system [26]. Therefore, additional study is needed to determine the optimum concentration of nanoparticles, nanoparticle size, and their distribution to produce the required mechanical characteristics without sacrificing the beneficial interaction between the scaffolds and the cell microenvironment. Also, it is difficult to produce high-quality nanofibers using this method due to their beaded appearance and inconsistent distribution of nanofibers. It may happen when nanoparticles are added to the solution, or gel contents exceed the solution’s threshold, which changes the solution’s surface tension and viscosity. It is still challenging to achieve reproducible fibers with centrifugal spinning for tissue engineering applications. The literature reports solution parameters but does not mention ambient apartments when fabricating centrifugal spinning-based fibers, even though it impacts reproducibility. In many situations, investigations also lack information on the state or layout of the apparatus and the relevant ambient conditions like temperature and humidity. Fabrication of anisotropic fibers remains challenging and restricts its applications as implants. Furthermore, there is a trade-off between improved cell adherence and increased enzymatic biodegradation when more of a particular biocompatible polymer content is added to the polymer blend [27]. Therefore, it is difficult to develop a binary or tertiary polymeric system that can work in a cell culture setting for a longer time. While an elevation in the gelatin percentage of a biocompatible polymer like this one may theoretically boost cell growth, the swelling properties would likely lower the material’s fibrillar character and permeability, decreasing cell survival [72].

## 8. Conclusions and Future Prospects

Over the last few decades, polymer nanofibers and their composites have become a highly significant material advancement. The centrifugal spinning process is increasingly being used to produce different types of polymer nano-fiber composite due to its low manufacturing cost and high production rate compared to other recognized approaches like electrospinning. Centrifugal spinning’s basic mechanism, solution, machine settings, and features are initially explored in this literature. Subsequently, we look at how the morphology itself could affect the performance, and properties of the tissue engineering application, considering factors such as fiber diameter, bead size, distribution, and alignment and how these factors are affected by the process and machine variables.

Many prospects exist for high-quality, large-scale production of centrifugally spun fibers, but this opens up a host of challenges, including machine modifications. Since the solution’s mass decreases as spinning continues, it’s challenging to keep the flow rate constant. Hence, some automated mechanisms for adjusting the spinneret’s rotational variables need to be developed to keep the jet’s outflow rate steady. The production of fibers without defects relies heavily on the rate of solvent evaporation, which is in turn heavily influenced by humidity. As such, a better design is necessary in order to maintain the ambient environment. In addition, it is crucial for fabricating tissue scaffolds that the fibers are appropriately aligned to facilitate the direction of cell growth along their axis. Also, new types of polymers and dielectric materials need to be investigated for the composite with high porosity without compromising its modulus. In this case, encouraging secondary bonds like electrostatic interactions and hydrogen bonding among the composite could be crucial for improving the final composite’s mechanical properties. However, in order to keep in conformal contact with skin or other organs, the stiffness of the nanofiber composite scaffold should be well-optimized. In many instances, spun fiber retains solvent following the spinning process. Therefore, the elimination of solvents is essential, and the development of alternative methods for doing so may yield useful insights into the composite’s pore region and morphologies.

Theoretical research or numerical modeling based on solution and machine characteristics is needed to predict the fibers’ diameter and distributions. A few pieces of literature demonstrate jet generation from a polymer solution using such models. However, it is unclear how the miscibility of various polymers influences the fiber characteristics, porosity, etc. Thereby, a wider variety of polymer blends may be exploited to learn about the multiphase behavior of polymer solutions, viscosity, and relevant fiber characteristics.

Numerous investigations have proven homopolymers as the foundation for the centrifugal method. Therefore, it is high time that the centrifugal spinning process is used to test copolymers in order to determine the features of fibers based on copolymers that may possess multifunctional properties. Several studies have used the centrifugal approach to examine VICs for potential use in tissue engineering applications involving heart valve tissues. However, research establishing the transition from valve endothelial cells to VICs should be investigated to comprehend endothelial to mesenchymal transition. Thus, it is necessary to manufacture more fiber-reinforced hydrogel-based composites so that this unexplored area may be probed. Very little study has been conducted on incorporating growth factors from biomolecules such as platelets into a nanofiber. There is still much room for further research into such biomolecules to establish the extent to which cell proliferation and metabolic activity may be enhanced in a vast array of biodegradable polymer composites.

## Figures and Tables

**Table 1:** Polymers used in the centrifugal spinning process, characteristics, and their applications in tissue engineering.

Polymers (P)	Solvent (S)	Molecular Weight (Mw) of Polymer	Rotational Speed (rpm)	Average Diameter (nm)	Weight Percentage, wt% (P/S)	Applications	Ref.
Polylactic acid (PLA)	Chloroform	-	4000/8000/12,000	1143/468/424	8/NA	Tissue engineering scaffolds	[28]
Hydroxypropyl cellulose	Distilled water	80,000		7 × 10^3^ to 20 × 10^3^	48	Formulation process of pharmaceutically active compound	[63]
Polyvinylpyrrolidone (PVP), Kollidon 30	Mixture of water & Ethanol (1:1)	30,000–50,000	-	(11 ± 3.7) ×10^3^	45/NA	conventional dosage forms or drug delivery systems	[82]
Polyvinylpyrrolidone (PVP)	Ethanol	1,300,000	15,000	(4.85 ± 1.37) × 10^3^ (16)	8, 10, 12, 14, 16, 18	Actives ingredients in drug delivery system	[56]
PVP/Iron Nitrate	Water	1,300,000	7000–7500	852 ± 86 (OD)	-	Catalyst, biological processes and biomedical applications	[83]
Ethyl cellulose (EC)/PVP	Ethanol & Water	NA/1300,000	3500	7000	-	Tissue engineering, drug delivery	[84]
Tannic acid (TA)/PVA/Chitosan (CS)	Distilled water	85,000–124,000/50,000–190,000	20,000	800	10	Wound dressing	[76]
PVA, Alginate di-aldehyde (ADA)/PVA	Deionized water	-	3000	139 ± 62, 258 ± 42	10	Skin tissue engineering	[36]
Polycaprolactone (PCL)	1,1,1,3,3,3-Hexafluoro-2-propanol (HFIP)	70,000–90,000	30,000	-	6 (*w*/*v*)	Tissue engineering	[21]
PCL	Dichloromethane, DMF, Chloroform	60,000, 80,000	9000, 4000	220 ± 98, 990	16,18	Neural tissue regeneration and tissue engineering	[33,75]
Nylon 6/Polyurethane (PU)	Dimethylformamide & Acetone	-	30,000	-	5/5	Flexible sensors, and drug-eluting materials	[55]
Nylon 6/Ag-Cu NPs	Formic Acid	-	8000	≥100	-	Wound dressing, tissue engineering	[85]
Polybutylene terephthalate (PBT)	Polymer Melt	-	10,000, 12,000, 15,000	1350, 1310, 1380	-	High-performance polymer fiber with desirable chemical/physical properties	[49]
Polyhydroxy butyrate (PHB)	Chloroform	550,000	5000–6000	1800	11	Tissue engineering	[86]
Chitosan (CS)	Trifluoroacetic acid & DCM	-	6000–9000	800 to 1500	7–9	Wound dressing	[13]
Teflon-AF (TAF) 1600	Fluorinert FC-40	-	5000–10,000	362 ± 58	10	Protective agent	[24]
Polystyrene (PS)	Dimethylformamide	260,000	5000–8000	9000, 5500, 3300, 2000	16, 18, 20, 22, 24	Superhydrophobic surface	[6]
Pullulan (PL)/Chondroitin Sulphate	Tannic acid (TA) & Citric acid (CA)	-	6500	400	18	Wound dressing	[87]
Poly(L-lactic acid) (PLLA)/PCL	Chloroform, Chloroform/Acetone	177,500/70,000–90,000	3450	1647 ± 976, 8002 ± 5051	6	Scaffold for cell growth	[88]
PU, Gelatin/PU	DMF	100,000	2800–3500	100 to 700, 2000 to 12,000	20	Dressing, scaffolding	[10]
Poly (vinyl chloride (PVC)	Cyclohexanon & DMF	230,000	3000	2000 to 12,000	16	Dressing, scaffolding	[10]
Lignin/Thermoplastic polyurethane (TPU)	DMF	-	6000–11,000	<500	15, 20, 25	Biomedical applications	[67]
Poly (3-hydroxybutyrate-*co*-3-hydroxyvalerate)(PHBV)	Chloroform	-	9000	500 to 3000	25	Biomedical applications	[73]
PLA melt	-	90,595	900	3470 to 3480	-	Tissue engineering	[29]
Chitosan/gelatin	Chloroform, DMF	190,000–310,000	10,000	1810	30	Soft tissue engineering	[41]
Poly(D,L-lactic aid)/ZnO/hydroxyapatite	Chloroform	160,000	400–600	780 ± 550 to 2390 ± 740	10	Bone tissue engineering	[26]
Silk fibroin (SF)/poly(ethylene oxide) (PEO)	Lithium bromide (for SF), Deionized water (for PEO)	200,000 (PEO)	36,000	710 to 2100	15 (PEO)	Bone tissue engineering	[89]
Poly (ethylene oxide)	Water	600,000	10,000	447 ± 165 to 596 ± 222	-	Cosmetic and dermatologic application	[40]
Polyvinylpyrrolidone/1-triacontene	-	-	7000–15,000	1000 to 2000	-	Adsorbent in cosmetic application	[90]
Polylactic-co-glycolic acid (PLGA)	Chloroform	-	-	-	1.5	Tissue engineering	[91]
Polydioxanone (PDO)	Deep eutectic solvents (DES)	-	700	10,000 to 20,000	-	Wound healing	[4]

## Data Availability

Not applicable.
